# Validation of Functional Lumen Imaging Probe Panometry Esophageal Motility Classification Version 2.0: A Study of 805 Patients

**DOI:** 10.1111/nmo.70188

**Published:** 2025-10-22

**Authors:** Ofer Z. Fass, Gaston Becherano, John E. Pandolfino, Wenjun Kou, Ashton C. Ellison, Chanakyaram A. Reddy, Anh D. Nguyen, Stuart J. Spechler, Vani J. A. Konda, Dustin A. Carlson

**Affiliations:** ^1^ Kenneth C. Griffin Esophageal Center of Northwestern Medicine, Department of Medicine, Division of Gastroenterology and Hepatology, Feinberg School of Medicine Northwestern University Chicago Illinois USA; ^2^ Baylor Scott and White Center for Esophageal Diseases, Division of Gastroenterology Baylor University Medical Center Dallas Texas USA

**Keywords:** achalasia, dysphagia, endoscopy, impedance, motility

## Abstract

**Background:**

Functional lumen imaging probe (FLIP) panometry assesses esophageal motility during sedated endoscopy. Recently, the FLIP panometry motility classification was refined (v2.0) in the Dallas Consensus. This study aimed to characterize esophageal motility using the FLIP v2.0 classification scheme and compare its classifications with those of FLIP v1.0 and high‐resolution manometry (HRM) diagnoses on the basis of Chicago Classification v4.0 (CCv4.0).

**Methods:**

805 adult patients who completed both FLIP and HRM at two tertiary esophageal centers were included; 704 with conclusive CCv4.0 diagnoses comprised the primary analysis, and 101 with inconclusive CCv4.0 diagnoses were also described. Esophagogastric junction (EGJ) opening and contractile response (CR) patterns were evaluated using 16‐cm FLIP during sedated endoscopy, with motility classifications defined by FLIP v1.0 and v2.0 criteria. HRM was classified per CCv4.0.

**Results:**

In the primary analysis, among the 137 (19%) patients with normal motility on FLIP panometry, 93% had normal motility or ineffective esophageal motility (IEM) on HRM. Normal motility on FLIP panometry had a 99% negative predictive value for disorders of EGJ outflow. Among the 163 (23%) patients with non‐spastic obstruction (defined by reduced EGJ opening and absent/diminished CR) on FLIP panometry, 91% had a conclusive disorder of EGJ outflow on HRM. Among the 37 patients with type III achalasia on HRM, 16 (43%) were classified as spastic obstruction on FLIP panometry.

**Conclusions:**

FLIP panometry v2.0 categorized esophageal motility in a manner that frequently paralleled the HRM/CCv4.0 diagnoses. FLIP panometry offers a well‐tolerated approach that complements, or may be an alternative to, HRM for diagnosing esophageal motility disorders.

## Graphical abstract

In a multicenter cohort of 805 patients, FLIP panometry v2.0 demonstrated strong diagnostic performance, with a 99% negative predictive value of normal motility for EGJ outflow disorders and a 91% positive predictive value of non‐spastic obstruction for achalasia or conclusive EGJOO, supporting its role as a valuable diagnostic tool.

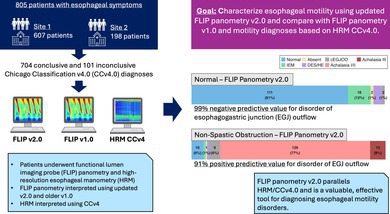

**Summary**

- FLIP v2.0 effectively categorizes esophageal motility in a manner that frequently aligns with HRM/CCv4.0.
- Normal motility on FLIP v2.0 demonstrates 99% negative predictive value for disorders of EGJ outflow and non‐spastic obstruction shows 91% positive predictive value for achalasia or conclusive EGJOO.
- FLIP panometry is a valuable and effective tool for diagnosing esophageal motility disorders.

## 1 Introduction

Functional lumen imaging probe (FLIP) panometry assesses esophageal motility by displaying esophageal diameter topography and associated distensive pressure in response to luminal distension [1]. Using a standardized volume distension protocol, FLIP panometry evaluates esophageal motility by analyzing esophagogastric junction (EGJ) opening mechanics and the contractile response (CR) to distension (i.e., secondary peristalsis), in an approach known as FLIP panometry [2]. Compared to esophageal high‐resolution manometry (HRM), FLIP offers advantages such as performance during sedated endoscopy (improving patient tolerance) and real‐time motility interpretation, with the procedure and interpretation requiring only several minutes to complete [3, 4].

**FIGURE 1:**
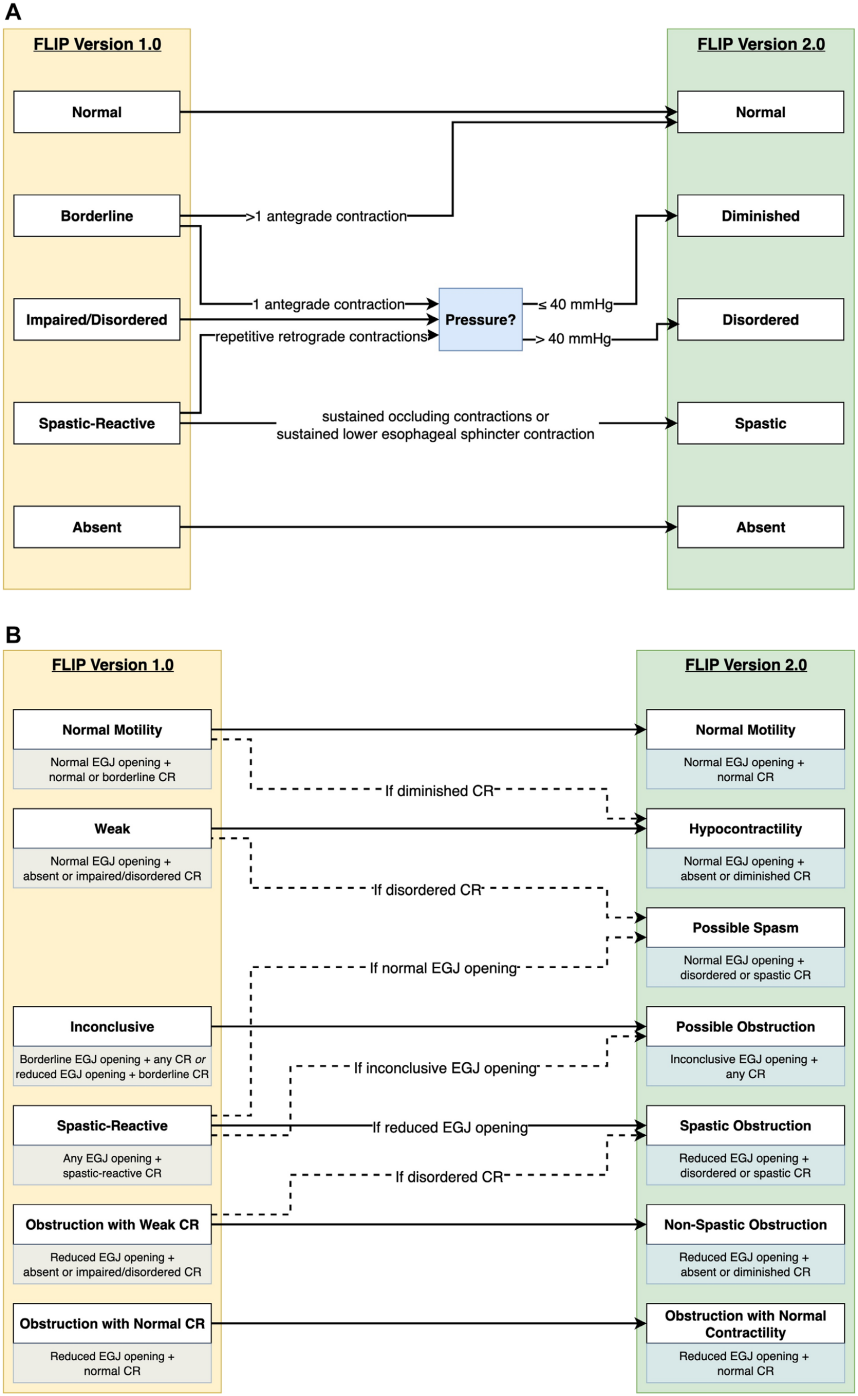
Summary of Changes from FLIP Panometry v1.0 to v2.0. A—Changes in Contractile Response Patterns. B—Changes in Motility Classifications. (A) The most significant revision in v2.0 involved contractile response (CR) patterns, with subdivision of impaired/disordered CR into diminished versus disordered CR on the basis of 60 mL pressure. (B) Changes in motility classification were primarily nomenclatural, with minor adjustments to CR and EGJ opening combinations. Motility classification criteria are noted below. Major updates are depicted with solid arrows, whereas minor changes are shown with dashed lines and explanations. The EGJ opening criteria remained unchanged, but the term “borderline EGJ opening” (v1.0) was replaced with “inconclusive EGJ opening” (v2.0). CR, contractile response; EGJ, esophagogastric junction.

The initial FLIP panometry classification scheme for esophageal motility (v1.0) demonstrated effectiveness in classifying esophageal motility in a manner comparable to HRM and the Chicago Classification version 4.0 (CCv4.0) in a cohort of 722 subjects [5]. Subsequent research and clinical experience led to the Dallas Consensus, which introduced FLIP panometry v2.0 that standardized the motility testing protocol and refined the classification scheme [6]. Although the criteria for EGJ opening remained unchanged aside from nomenclature, v2.0 introduced updates to contractile response patterns and motility classifications. Notably, the impaired/disordered contractile response category in v1.0 was subdivided into diminished versus disordered contractile response using a 40 mmHg pressure threshold at 60 mL, and obstruction with weak contractile response was reclassified into non‐spastic and spastic subtypes to improve achalasia phenotyping; Figure 1 [5, 6]. These updates were intended to enhance alignment between FLIP and HRM findings. This refinement mirrors the iterative changes of the Chicago Classification for the diagnosis of esophageal motility disorders with HRM [7].

In light of these refinements, this study aimed to characterize esophageal motility in a multicenter patient cohort using the updated FLIP panometry v2.0 classification scheme and compare its classifications with those of FLIP panometry v1.0 as well as motility diagnoses on the basis of HRM and CCv4.0 [7].

## 2 Methods

### 2.1 Subjects

Adult subjects (ages 18–91 years) presenting to the Esophageal Center of Northwestern Medicine (site 1) and to the Baylor Scott and White Center for Esophageal Diseases (site 2) for esophageal symptom evaluation between January 2020 and May 2024 were consecutively enrolled in site‐specific esophageal motility registries. Subjects who underwent FLIP during sedated esophagogastroduodenoscopy (EGD) with corresponding HRM were included in this analysis. Timed barium esophagram (TBE) was performed at the treating gastroenterologist's discretion when clinically indicated. No endoscopic or surgical interventions occurred between FLIP, HRM, and TBE studies. Subjects with technically limited FLIP or HRM studies were excluded. Additionally, those with prior foregut surgery (including pneumatic dilation) or esophageal mechanical obstruction (esophageal stricture, eosinophilic esophagitis, severe reflux esophagitis [Los Angeles grade C or D], or hiatal hernia > 3 cm) were excluded, as these conditions can cause secondary esophageal motor abnormalities. The cohort in this study is distinct from the 722 subjects included in the previous FLIP panometry motility classification studies that reflected the v1.0 classification scheme, which analyzed patients from 2012 to 2019 [5].

### 2.2 FLIP Study Protocol and Analysis

FLIP studies were performed during sedated EGD using a 16‐cm FLIP catheter (EndoFLIP EF‐322 N; Medtronic, Shoreview, MN) per the Dallas consensus protocol [6, 8, 9]. EGD was conducted with patients in the left lateral decubitus position using moderate sedation (midazolam and fentanyl) or propofol (and other medications possible) during monitored anesthetic care at the anesthetist's discretion. Previous research demonstrated that endoscopic sedation choice (i.e., moderate sedation vs. propofol) does not meaningfully impact FLIP motility findings relative to HRM diagnosis [10]. Following endoscope withdrawal and calibration to atmospheric pressure, the FLIP catheter was inserted transorally and positioned with the distal 1–3 impedance sensors located beyond the EGJ. This positioning was maintained throughout the study. The FLIP bag was filled in stepwise 10‐ml increments from 40 mL to a target volume of 70 mL, with each distension volume maintained for 30–60 s following stepwise filling.

FLIP data were analyzed post‐endoscopy using a customized program (available at http://www.wklytics.com/nmgi) by raters (site 1: D.A.C. and J.E.P.; site 2: G.B., A.C.E., and V.J.A.K.) who were blinded to clinical details, including HRM and TBE results [11]. Consensus was achieved at each site on all interpretations. Interpretation utilized FLIP Panometry motility classification v1.0 (Table S1) and v2.0 (Table 1) [5, 6]. Both schemes assessed the contractile response pattern during the entire study period from 50 to 70 mL fill volumes and EGJ opening classification on the basis of EGJ‐distensibility index at 60 mL fill volume and maximum EGJ diameter at 60–70 mL [12, 13]. The contractile response pattern and EGJ opening classification were then applied to assign a FLIP Panometry motility classification [2, 5, 6].

**TABLE 1:** Classification of esophageal motility with FLIP Panometry version 2.0.

FLIP Panometry CR patterns	Definition
Normal contractile response	Multiple (> 1) distinct antegrade contractions of at least 6‐cm axial length associated with a FLIP pressure rise > 10 mmHg With or without a repetitive antegrade contraction pattern Not meeting criteria for spastic contractile response
Diminished contractile response	Contractility present, but not meeting criteria for normal or spastic contractile response FLIP pressure (with 60 mL EGJ‐DI) ≤ 40 mmHg
Disordered contractile response	Contractility present, but not meeting criteria for normal or spastic contractile response FLIP pressure (with 60 mL EGJ‐DI) > 40 mmHg
Absent contractile response	No contractile activity in the esophageal body
Spastic contractile response	Presence of any of the following features: Sustained occluding contractions *or* Sustained lower esophageal sphincter contractions

FLIP Panometry EGJ opening classifications	
Reduced EGJ opening	EGJ‐DI < 2.0 mm^2^/mmHg *and* Maximum EGJ diameter < 12 mm
Inconclusive EGJ opening	EGJ‐DI < 2.0 mm^2^/mmHg OR Maximum EGJ diameter < 16 mm, Not meeting criteria for “reduced EGJ opening”
Normal EGJ opening	EGJ‐DI ≥ 2.0 mm^2^/mmHg *and* Maximum EGJ diameter ≥ 16 mm

Motility classification	EGJ opening classification	Contractile response pattern
*FLIP Panometry motility classification, v2.0*		
Normal motility	Normal	Normal
Hypocontractility	Normal	Absent or Diminished
Possible spasm	Normal	Spastic or Disordered
Possible obstruction	Inconclusive	Any^a^
Spastic obstruction	Reduced	Spastic or Disordered
Obstruction with normal contractility	Reduced	Normal
Non‐spastic obstruction	Reduced	Absent or Diminished

*Note:* The contractile response (CR) to distension was based on the evaluation of the functional lumen imaging probe (FLIP) study protocol including from the 50‐ to 70‐mL fill volume. EGJ opening applied the EGJ‐DI from the 60‐mL FLIP fill volume and the maximum EGJ diameter from the 60‐ or 70‐mL FLIP fill volume.

^a^ It is recommended to specify the CR pattern associated with a possible obstruction motility classification.

### 2.3 HRM Protocol and Analysis

Following a minimum 6‐h fast, HRM studies were conducted using a 4.2‐mm outer diameter solid‐state assembly with 36 circumferential pressure sensors at 1‐cm intervals (Medtronic, Shoreview, MN). The HRM assembly was placed transnasally and positioned to record from the hypopharynx to the stomach, with approximately three intragastric sensors included. After a 2‐min baseline recording, the CCv4.0 HRM protocol was performed, consisting of 10 5‐mL liquid swallows in the supine position followed by five 5‐mL liquid swallows in the upright position [7]. Studies were analyzed according to the CCv4.0 criteria, with investigators blinded to clinical characteristics, including FLIP and TBE results. A median integrated relaxation pressure (IRP) of > 15 mmHg was considered abnormal for supine swallows, whereas a median IRP of > 12 mmHg was considered abnormal for upright swallows.

In cases of inconclusive HRM diagnosis (i.e., EGJ outlet obstruction [EGJOO] or absent contractility with IRP at the upper limit of normal), TBE was applied to further classify the findings [7, 14, 15]. TBE was conducted in the upright position and involved consuming 200 mL of low‐density barium sulfate, with anteroposterior images obtained at 1, 2, and 5 min [16]. A 12.5 mm barium tablet was administered if there was not significant retention after 5 min. An abnormal TBE, defined by a barium column height > 5 cm at 5 min or a barium column height > 5 cm at 1 min in addition to a tablet impaction, was applied to define *conclusive* disorders of EGJ outflow (among inconclusive HRMs). A normal TBE indicated normal EGJ outflow. A missing or inconclusive TBE (defined as either: barium column height > 5 cm at 1 min and ≤ 5 cm at 5 min with no tablet administration or tablet passage; or barium height ≤ 5 cm at 1 min with tablet impaction) was interpreted as *inconclusive* EGJ outflow [17]. Inconclusive HRM/CCv4.0 results were excluded from the primary analysis because of the clinical uncertainty of these HRM and/or TBE findings; these cases were assessed in the secondary analysis.

## 3 Statistical Analysis

Results were reported as mean (standard deviation; SD) or median (interquartile range; IQR), depending on data distribution. Group comparisons were performed using the *χ*2 test for categorical variables and analysis of variance/*t* tests or Kruskal–Wallis/Mann–Whitney *U* test for continuous variables, on the basis of data distribution. Statistical significance was set at a two‐tailed *p* value of < 0.05. Data analysis was conducted using R version 4.4.2 [18]. Data cleaning and reshaping were performed using the tidyverse package [19]. Data visualization, including bar plots and contingency tables, was completed using the ggplot2 package [20].

## 4 Results

### 4.1 Subjects

The study cohort comprised 805 patients with a mean age of 55 (17) years; 62% were female; Table 2. A total of 607 subjects were evaluated at site 1 (Table S2) and 198 at site 2 (Table S3). Dysphagia was the most common indication for motility evaluation (75%). Most participants (66%) underwent FLIP and HRM on the same day, whereas the remainder had a median interval of 1.5 (0.6–3.2) months between studies. The most frequent HRM classifications were normal esophageal motility in 264 (33%) and achalasia (subtypes I, II, or III) in 241 individuals (30%). The subset of 101 patients with inconclusive HRM/CCv4.0 diagnoses had a mean age of 61 (14) years, with 66% being female; Table S4. The most common reason for an inconclusive diagnosis was an inconclusive HRM (typically EGJOO) and the absence of a TBE (88 participants). No adverse events occurred during FLIP studies.

**TABLE 2:** Cohort characteristics.

	Total *n* = 805	FLIP panometry v2.0 motility classification					
		Normal *n* = 156	Hypo‐contractility *n* = 84	Non‐spastic obstruction *n* = 174	Spastic obstruction *n* = 96	Possible spasm *n* = 62	Possible obstruction *n* = 233
*Demographics*							
Age, mean (SD)	55 (17)	45 (16)	54 (16)	52 (18)	64 (14)	59 (15)	59 (15)
Sex, female	495 (62)	112 (72)	59 (70)	76 (44)	51 (53)	52 (84)	145 (63)
Indication							
Dysphagia	604 (75)	100 (64)	57 (68)	144 (83)	86 (90)	38 (61)	179 (77)
Reflux symptoms	64 (8.0)	34 (22)	7 (8.3)	4 (2.3)	2 (2.1)	6 (9.7)	11 (4.7)
Chest pain	35 (4.3)	5 (3.2)	5 (6.0)	3 (1.7)	3 (3.1)	6 (9.7)	13 (5.6)
Other	89 (11)	14 (9.0)	13 (16)	20 (12)	5 (5.2)	13 (21)	24 (10)
*Endoscopy*							
Esophagitis							
LA grade A	30 (3.7)	10 (6.4)	7 (8.3)	1 (0.6)	2 (2.1)	3 (4.8)	7 (3.0)
LA grade B	17 (2.1)	4 (2.6)	7 (8.3)	0 (0)	0 (0)	1 (1.6)	5 (2.1)
Non‐obstructing ring	20 (2.5)	8 (5.1)	2 (2.4)	0 (0)	7 (7.3)	0 (0)	3 (1.3)
Diverticulum	22 (2.7)	0 (0)	1 (1.2)	6 (3.4)	5 (5.2)	5 (8.1)	5 (2.1)
*Manometry*							
Conclusive CCv4.0 Diagnosis							
Normal	264 (33)	111 (71)	21 (25)	10 (5.7)	13 (14)	33 (53)	76 (33)
IEM	88 (11)	16 (10)	22 (26)	2 (1.1)	4 (4.2)	12 (19)	32 (14)
Absent	45 (5.6)	2 (1.3)	34 (40)	1 (0.6)	2 (2.1)	1 (1.6)	5 (2.1)
DES	9 (1.1)	2 (1.3)	0 (0)	0 (0)	1 (1.0)	0 (0)	6 (2.6)
Hypercontractile	29 (3.6)	4 (2.6)	0 (0)	2 (1.1)	8 (8.3)	4 (6.5)	11 (4.7)
EGJOO	28 (3.5)	2 (1.3)	0 (0)	9 (5.2)	7 (7.3)	0 (0)	10 (4.3)
Type I achalasia	72 (8.9)	0 (0)	0 (0)	50 (29)	2 (2.1)	1 (1.6)	19 (8.2)
Type II achalasia	132 (16)	0 (0)	1 (1.2)	76 (44)	22 (23)	0 (0)	33 (14)
Type III achalasia	37 (4.6)	0 (0)	0 (0)	13 (7.5)	16 (17)	0 (0)	8 (3.4)
Inconclusive CCv4.0 Diagnosis							
Inconclusive EGJOO	98 (12)	19 (12)	6 (7.1)	9 (5.2)	21 (22)	11 (18)	32 (14)
Inconclusive	3 (0.4)	0 (0)	0 (0)	2 (1.1)	0 (0)	0 (0)	1 (0.4)
HRM‐EGJ Morphology							
Type I (no HH)	509 (63)	79 (51)	37 (44)	149 (86)	58 (60)	34 (55)	152 (65)
Type II‐III (HH)	247 (31)	72 (46)	45 (54)	15 (8.6)	25 (26)	24 (39)	66 (28)

*Note:* Values represent *n* (%) unless otherwise specified.

Abbreviations: CCv4.0, Chicago Classification v4.0; DES, distal esophageal spasm; EGJ, esophagogastric junction; EGJOO, esophagogastric junction outflow obstruction; HH, hiatal hernia; HRM, high‐resolution esophageal manometry; IEM, ineffective esophageal motility.

### 4.2 FLIP Panometry Evaluation

**FIGURE 2:**
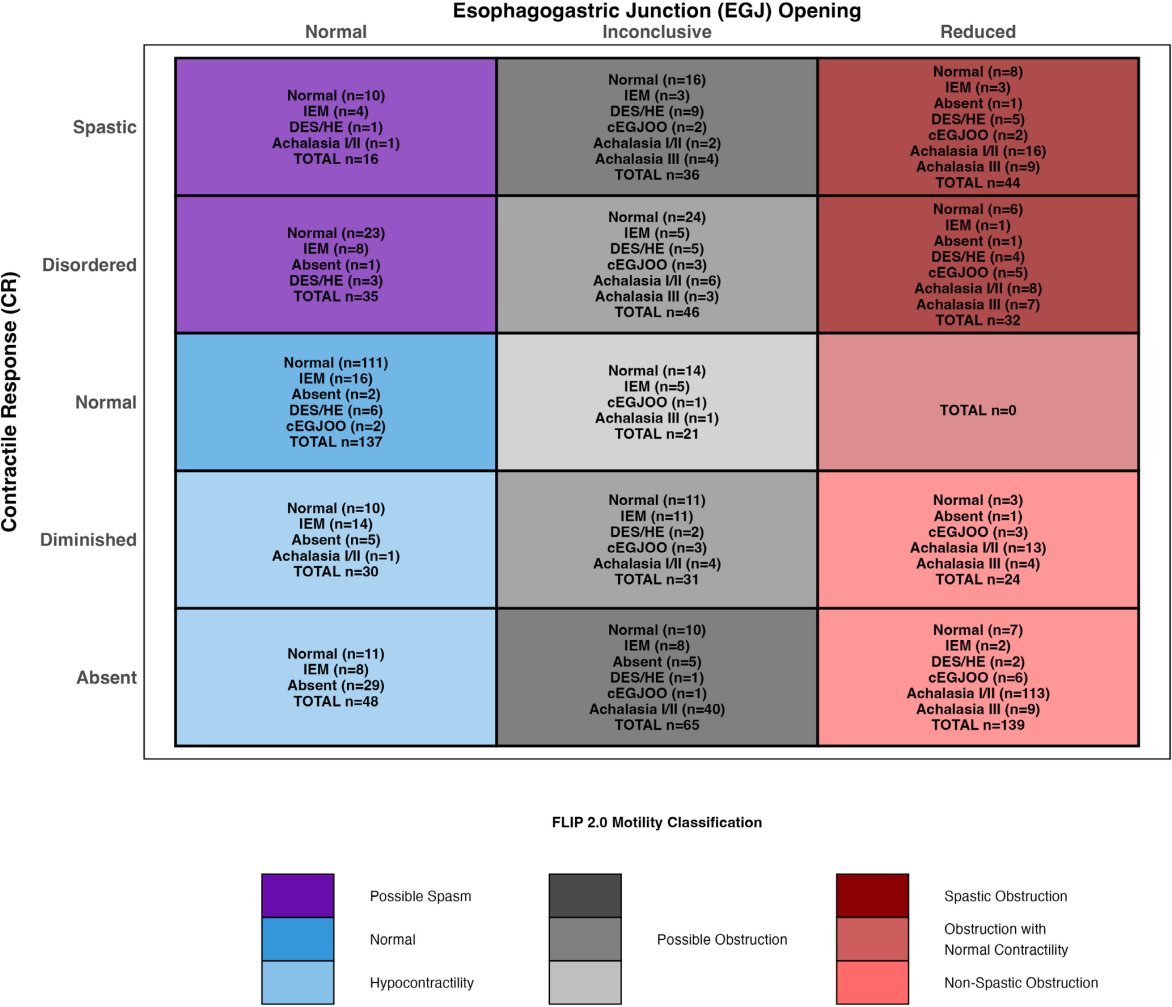
Distribution of Chicago Classification Diagnoses Across FLIP Panometry v2.0 Categories. Distribution of CCv4.0 diagnoses across FLIP panometry v2.0 metrics as a matrix plot with EGJ opening plotted against contractile response. Within each cell is the total number of patients and their CCv4.0 diagnoses. Color coding represents the corresponding FLIP v2.0 classifications. CCv4.0, Chicago Classification v4.0; DES, distal esophageal spasm; EGJOO, esophagogastric junction outflow obstruction; HE, hypercontractile esophagus; IEM, ineffective esophageal motility.

In the primary analysis of 704 participants with a conclusive HRM/CCv4.0 diagnosis, FLIP panometry classifications of EGJ opening included 266 participants (38%) with normal EGJ opening, 199 (28%) with inconclusive EGJ opening, and 239 (34%) with reduced EGJ opening; Figure 2. Among 266 patients with normal EGJ opening, 98.5% had normal EGJ outflow per HRM/CCv4.0, whereas 81.6% of the 239 patients with reduced EGJ opening had a disorder of EGJ outflow. FLIP panometry CR patterns comprised 158 (22.4%) participants with normal CR, 85 (12.1%) with diminished CR, 252 (35.8%) with absent CR, 113 (16.1%) with disordered CR, and 96 (13.6%) with spastic CR. Only one patient with normal CR had achalasia on HRM (which was type III achalasia), and three had conclusive EGJOO.

### 4.3 FLIP Panometry v2.0 Motility Classifications

**FIGURE 3:**
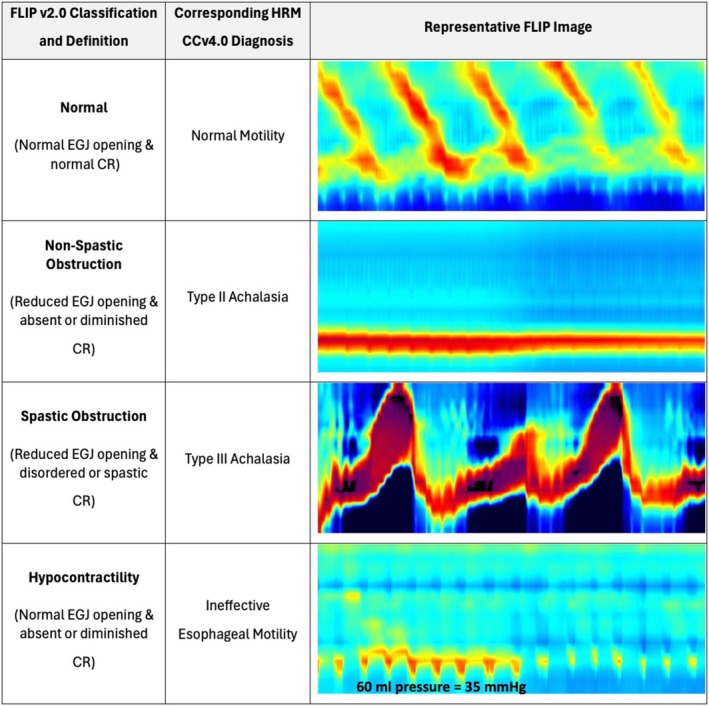
Representative FLIP Panometry v2.0 Patterns and Corresponding High‐Resolution Manometry Diagnoses. Each image is 1 min in length and includes the study during the 60–70 mL fill volumes. Normal panometry correlates with normal motility, demonstrated by more than 1 antegrade contraction present and normal EGJ opening. Non‐spastic obstruction (typical of type I or II achalasia) demonstrates reduced EGJ opening with minimal or absent contractile activity in the esophageal body. Spastic obstruction (representative of type III achalasia) exhibits reduced EGJ opening with disordered and spastic contractile response. Hypocontractility (corresponding to ineffective esophageal motility) shows normal EGJ opening with diminished or absent contractility in the esophageal body. CCv4.0, Chicago Classification v4.0; CR, contractile response; EGJ, esophagogastric junction; HRM, high‐resolution manometry.

The FLIP panometry v2.0 classifications of esophageal motility in the total cohort included normal motility in 19% of individuals, hypocontractility in 11%, non‐spastic obstruction in 23%, spastic obstruction in 11%, possible spasm in 7.2%, and possible obstruction in 28%; Table 2. No patients were classified as obstruction with normal contractility. The representative examples in Figure 3 illustrate the characteristic FLIP panometry patterns of normal motility, non‐spastic obstruction, spastic obstruction, and hypocontractility with their corresponding HRM diagnoses.

Dysphagia was the most common indication for motility testing across all FLIP v2.0 classifications, whereas reflux was more commonly reported in those with normal, hypocontractility, and possible spasm classifications compared to other FLIP v2.0 categories. Mild esophagitis (Los Angeles Grade A or B) was more common in normal and hypocontractility classifications than in non‐spastic obstruction and spastic obstruction. Hiatal hernia (on HRM; ≤ 3 cm) was least frequently observed in patients with non‐spastic obstruction.

**FIGURE 4:**
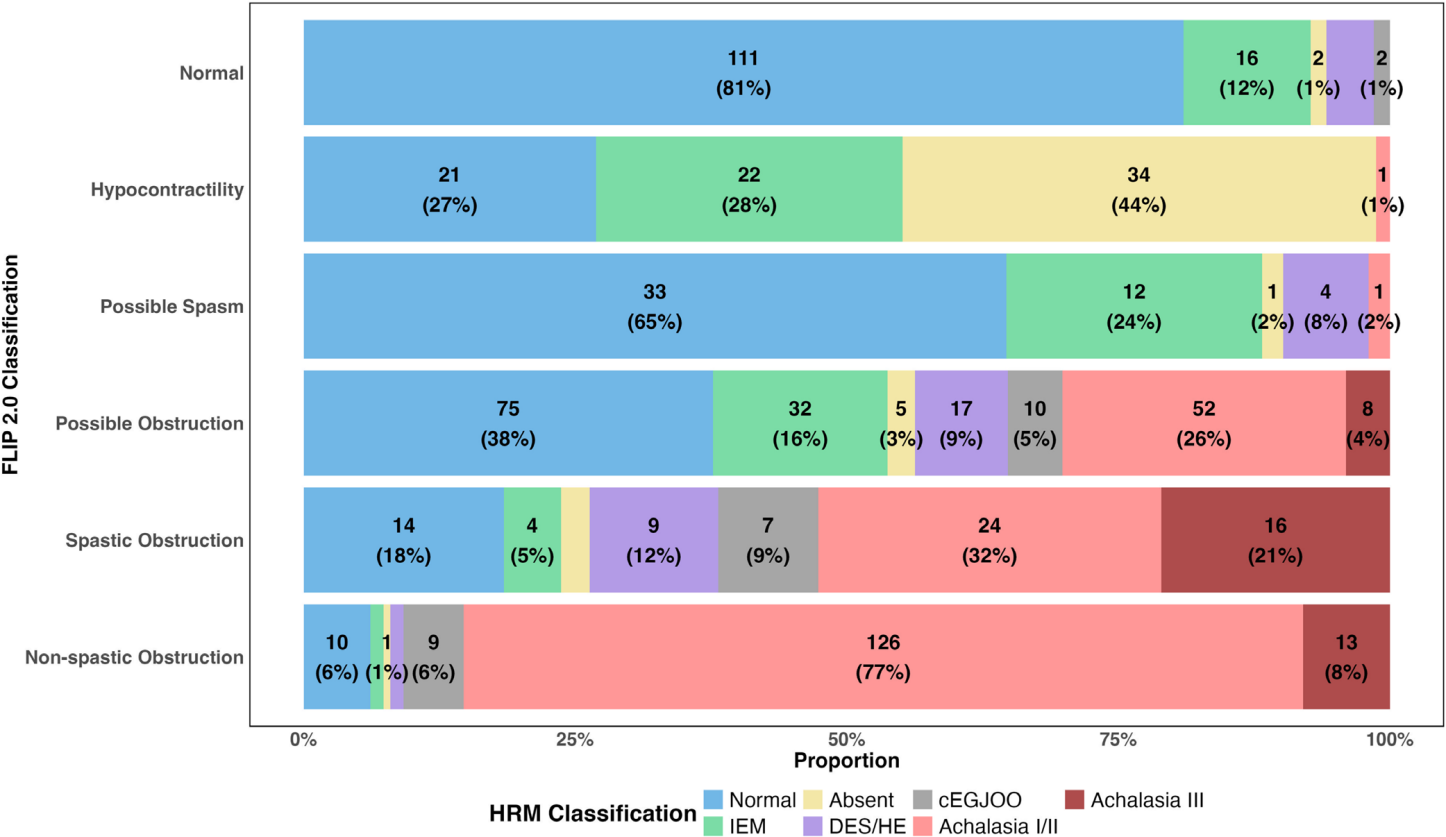
Distribution of Chicago Classification Diagnoses Across FLIP Panometry v2.0 Classifications. Stacked bar plots displaying the proportion of CCv4.0 diagnoses within each FLIP panometry v2.0 classification category. CCv4.0, Chicago Classification v4.0; DES, distal esophageal spasm.

Among the 137 patients classified as normal by FLIP panometry, 81% had normal motility and 12% had ineffective esophageal motility (IEM) per CCv4.0; Figure 4. Normal motility on FLIP also demonstrated a 99% negative predictive value for disorders of EGJ outflow, with only 2 patients having conclusive EGJOO and none having achalasia on HRM. Among the 78 classified as hypocontractility, 72% had either absent contractility or IEM, whereas 27% had normal motility. One patient with hypocontractility on FLIP panometry had type II achalasia, though treatment was deferred as symptoms were minimal and TBE had normal clearance by 1 min with no progression of symptoms on follow‐up after 1 year.

Among 163 patients with non‐spastic obstruction, 77% had type I (31%) or II (47%) achalasia, 8.0% had type III achalasia, and 5.5% had conclusive EGJOO. In total, 91% of non‐spastic obstruction had a disorder of EGJ outflow by HRM/CCv4.0. Of the 76 patients with spastic obstruction, 32% had type I (2.6%) or II (29%) achalasia, and 21% had type III achalasia. Among the 37 patients with type III achalasia, 43% were classified as having spastic obstruction, 35% as non‐spastic obstruction, and 22% as possible obstruction. Among the 199 patients classified as possible obstruction, 38% had normal motility, 26% had type I/II achalasia, and 16% had IEM.

### 4.4 Comparing FLIP Panometry v1.0 and v2.0

**FIGURE 5:**
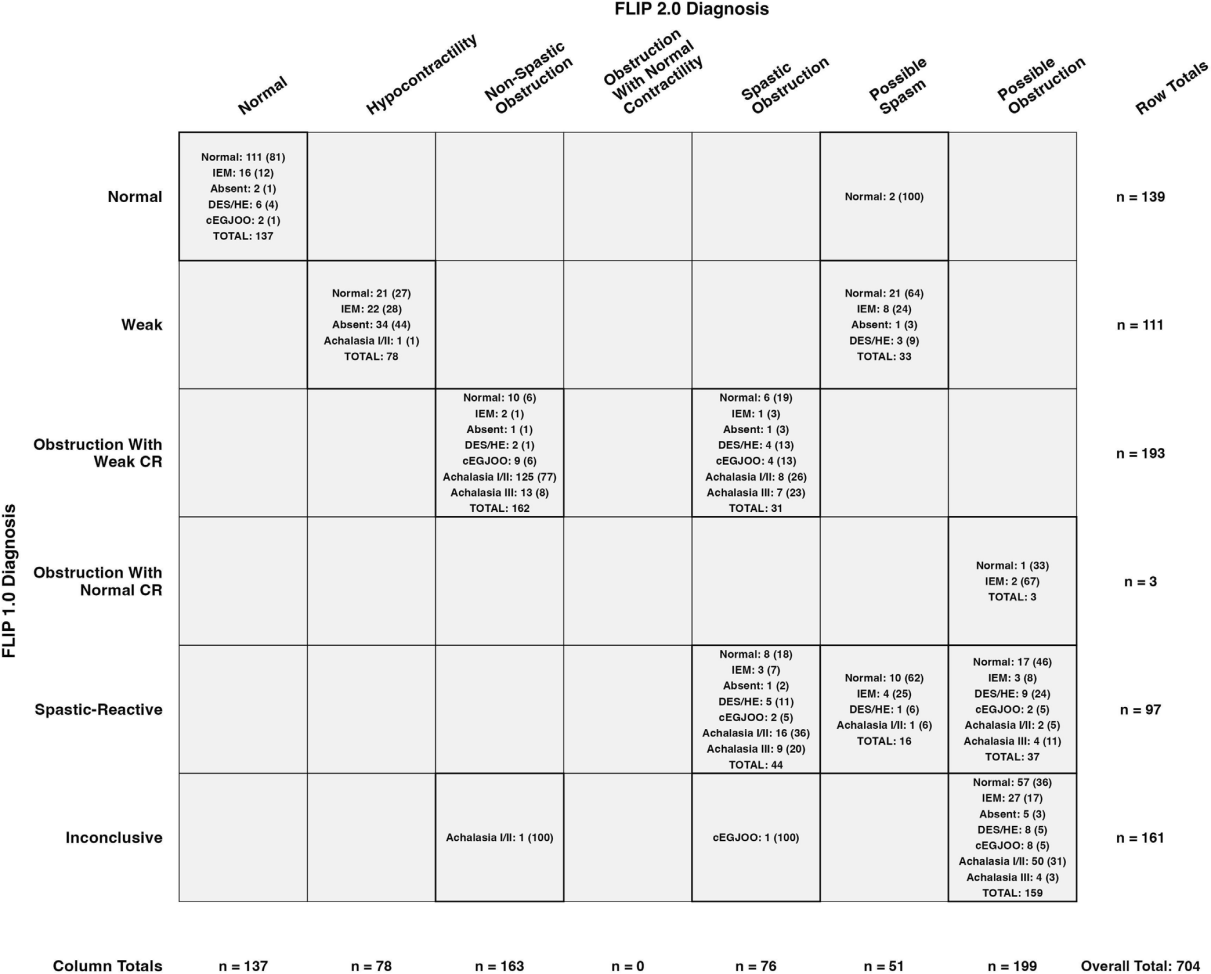
Contingency table of FLIP v1.0 versus FLIP v2.0 classifications. Corresponding Chicago Classification v4.0 high‐resolution manometry diagnoses are included within each cell, with cell displays counts as *n* (%), where the percentage represents the proportion within the respective cell. CR, contractility; DES, distal esophageal spasm; cEGJOO, conclusive esophagogastric junction outflow obstruction; HE, hypercontractile esophagus; IEM, ineffective esophageal manometry.

A contingency table comparing HRM/CCv4.0 classifications across FLIP panometry v1.0 and v2.0 is shown in Figure 5, noting changes in criteria from v1.0 to v2.0 (Figure 1). Patients classified as having obstruction with weak CR in FLIP panometry v1.0 (of whom 26% had type I achalasia, 43% type II achalasia, and 10% type III achalasia) were reclassified using FLIP v2.0 as either non‐spastic obstruction (31% type I achalasia, 47% type II achalasia, and 8.0% type III achalasia) or spastic obstruction (3% type I achalasia, 29% type II achalasia, and 21% type III achalasia). Among patients with a weak classification by FLIP panometry v1.0, 32% had absent contractility, 27% had IEM, and 2.7% had hypercontractile esophagus. When reclassified using FLIP panometry v2.0, these patients were categorized as having either hypocontractility (44% absent contractility, 28% IEM, 0% hypercontractile or spasm) or possible spasm (2.0% absent contractility, 24% IEM, 7.8% hypercontractile esophagus, 0% spasm). Finally, among patients classified as spastic reactive by FLIP panometry v1.0, 13% had type III achalasia. When reclassified using FLIP v2.0, these patients were categorized as having spastic obstruction (21% of type III achalasia), possible obstruction (4.0% of type III achalasia), or possible spasm (0% type III achalasia); Figure 5.

## 5 Discussion

This multicenter study of 805 patients demonstrated that FLIP panometry v2.0 motility classifications effectively categorize esophageal motility in a manner that frequently aligns with motility evaluation using HRM and CCv4.0. Normal motility on FLIP was associated with normal motility or IEM on HRM in 127 (93%) patients, whereas non‐spastic obstruction on FLIP was associated with a disorder of EGJ outflow per HRM/CCv4.0 in 148 patients (91%), including 77% with non‐spastic (i.e., type I or II) achalasia. Overall, these results continue to support the effectiveness of FLIP Panometry for the diagnosis of esophageal motility disorders.

With a minor change in criteria for normal motility on FLIP panometry from v1.0 to v2.0 (Figure 1), normal motility on FLIP panometry demonstrated a high negative predictive value (99%) for disorders of EGJ outflow per HRM/CCv4.0, including 0 patients with achalasia, consistent with previous findings using FLIP v1.0 [5, 6]. Although not all patients with normal motility on FLIP had normal motility on HRM, the proportion of patients with distal esophageal spasm (DES) and IEM observed among patients with normal motility on FLIP v2.0 aligns with the proportions of DES (1%–14%) and IEM (7%–17%) observed in healthy, asymptomatic volunteers undergoing HRM [21]. Hence, the present study provides additional evidence that normal motility on FLIP panometry can effectively rule out achalasia and, if assessed during index endoscopy, can guide management toward reflux or a functional syndrome without necessitating HRM as a subsequent diagnostic step.

Furthermore, the FLIP panometry v2.0 category of non‐spastic obstruction, which corresponds to obstruction with weak CR in FLIP panometry v1.0, remained strongly associated with achalasia or conclusive EGJOO, with a positive predictive value of 91%. A major update in FLIP v2.0 was the differentiation of the impaired/disordered contractile response (IDCR) in v1.0 into diminished CR or disordered CR. This distinction is based on the 60 mL FLIP pressure measurements from a study demonstrating that spasm occurred more frequently in high‐pressure IDCR cases but could be effectively ruled out in low‐pressure scenarios [22]. Accordingly, a pressure threshold of 40 mmHg at the 60‐mL EGJ‐DI was incorporated into the FLIP v2.0 criteria to differentiate disordered from diminished CR patterns in patients who do not meet criteria for normal, spastic, or absent CR. This refinement appeared to improve subtyping of achalasia (i.e., differentiating non‐spastic from spastic) compared with FLIP panometry v1.0. Non‐spastic obstruction with v2.0 (which includes diminished CR, as well as absent CR) was 77% non‐spastic (i.e., type I or II) achalasia, compared with 69% of type I/II achalasia with obstruction with weak CR on v1.0; Figure 5. Further, the v2.0 spastic obstruction classification (which includes disordered CR, as well as spastic CR) encompassed nearly half of all type III achalasia patients in this cohort (16 patients), comprising 21% of spastic obstruction cases on FLIP. Notably, this group also included 22 patients (29%) with type II achalasia and only 2 patients (2.6%) with type I achalasia, noting that a spastic variant of type II achalasia may be identifiable using FLIP Panometry [23]. These findings parallel our prior work, which showed that elevated FLIP 60 mL pressures and spastic‐reactive contractile responses were associated with post‐treatment spasm in type II achalasia (while also noting the uncertain clinical relevance of this finding), whereas absent contractility was more frequent in those without spasm [24]. In contrast, when using FLIP v1.0 classifications, type III achalasia patients were predominantly divided between obstruction with weak CR (20 patients, 10% of the classification) and spastic‐reactive classifications (13 patients, 13% of the classification).

Further, refinement of IDCR from v1.0 to include the diminished CR pattern in FLIP v2.0 appeared to enhance the identification of hypocontractile disorders. Specifically, 72% of those classified as hypocontractility by FLIP v2.0 had an HRM/CCv4.0 diagnosis of IEM or absent contractility, compared to 59% using the FLIP v1.0 classification of weak motility (in part defined by IDCR). A trade‐off in FLIP v2.0, however, is the introduction of the “possible spasm” classification, intended to distinguish primary esophageal spasm from hypocontractile disorders. Although only 8% of patients classified as “possible spasm” had hypercontractile esophagus or DES per HRM/CCv4.0, this was a higher proportion compared to other non‐obstructive FLIP v2.0 classifications. In contrast, 65% of patients with “possible spasm” had normal HRM, which, given the rarity of DES, suggests impaired secondary peristalsis rather than a spastic variant with normal HRM, though further study of this subgroup is warranted. Although the rarity of DES should be noted, the possible spasm classification of FLIP Panometry v2.0 could identify patients with a spastic esophageal motility disorder, warranting further evaluation with HRM. Ultimately, the “possible spasm” classification remains an area that requires further refinement and validation.

Strengths of this study include the use of a large patient cohort with comprehensive esophageal evaluations, the application of HRM and CCv4.0, and the inclusion of a cohort distinct from the initial FLIP panometry v1.0 study [5]. The multicenter design enhances the generalizability of the findings beyond single‐center studies, though the two esophageal referral centers may not reflect patient cohort characteristics of community practices. The higher prevalence of achalasia compared to community settings may represent a strength, as achalasia identification is the most clinically significant outcome of esophageal motility testing, and EGJOO represents an important yet clinically challenging diagnostic pattern.

A limitation of this study, which represents a key challenge of esophageal motility research more broadly, is the absence of a definitive gold standard for diagnosis. Despite using state‐of‐the‐art methods, including HRM and CCv4.0 with TBE, inconclusive and discordant diagnoses still occur. These inconclusive results limit the evaluation of FLIP panometry, as an independent, conclusive diagnosis is necessary to objectively assess its performance. That said, the goal of FLIP is not to replicate HRM, as the two methods are related, yet assess distinct physiological processes (i.e., HRM evaluates primary peristalsis, whereas FLIP assesses secondary peristalsis). Recognizing the potential discordance between HRM, FLIP, and TBE highlights the need for complementary diagnostic approaches and underscores opportunities for future research to clarify the clinical significance and application of discordant test results [25].

In conclusion, FLIP panometry classification using the updated FLIP panometry v2.0 scheme from the Dallas consensus accurately identified esophageal motility disorders, including achalasia, in this novel multicenter cohort [6]. FLIP panometry should be interpreted in the context of endoscopic and clinical data, similar to HRM. Because FLIP panometry is performed during sedated EGD, it can enhance the diagnostic yield of the endoscopy encounter. When applied during the index examination, FLIP has the potential to expedite diagnosis of esophageal motility disorders; in other scenarios, it may inform immediate endoscopic interventions, such as reinspection or dilation, or guide the need for further testing when results are abnormal or inconclusive [26]. Although FLIP complements HRM, in select cases, it may serve as a viable alternative. Future studies will explore the application of FLIP Panometry v2.0 in broader populations, assess longitudinal outcomes, and further define unique subgroups of interest. As with HRM and the evolving Chicago Classification, this diagnostic paradigm will continue to develop with growing clinical experience and research. Overall, these findings further validate FLIP panometry as a valuable and effective tool for diagnosing esophageal motility disorders.

## Author Contributions

O.Z.F. contributed to data analysis, visualization, interpretation, drafting of the manuscript, and approval of the final version. G.B. contributed to data analysis, interpretation, and approval of the final version. J.E.P. contributed to obtaining funding, data interpretation, editing the manuscript critically, and approval of the final version. A.C.E. contributed to data collection, interpretation, and approval of the final version. A.C.E., C.A.R., A.D.N., and S.J.S. contributed to data collection, interpretation, and approval of the final version. W.K. contributed to data collection, analysis, and approval of the final version. V.J.A.K. contributed to the study concept, data collection, editing the manuscript, and approval of the final version. D.A.C. contributed to the study concept and design, drafting of the manuscript, obtaining funding, data analysis, data interpretation, and approval of the final version.

## Conflicts of Interest

J.E.P.: Sandhill Scientific/Diversatek (Consulting, Grant), Takeda (Speaking), Astra Zeneca (Speaking), Medtronic (Speaking, Consulting, Patent, License), Torax/Ethicon (Speaking, Consulting), EndoGastric Solutions (Advisory Board), Phathom (Speaking, Consulting). W.K.: BMS (Consulting). S.J.S. has served as a consultant for Takeda, Phathom Pharmaceuticals. V.J.A.K.: Medtronic (Consulting), Ambu (Consulting), Braintree (Consulting), Exact Sciences (Consulting, Advisory Board), Castle (Speaking). D.A.C.: Medtronic (Speaking, Consulting, License); Diversatek (Consulting); Braintree (Consulting); Medpace (Consulting); Phathom Pharmaceuticals (Speaking, Consulting); Regeneron/Sanofi (Speaking). Other authors declare no conflicts of interest.

## Supporting information


**Appendix S1:** nmo70188‐sup‐0001‐AppendixS1.docx.

## Data Availability

The data that support the findings of this study are available from the corresponding author upon reasonable request and completion of necessary privacy and ethical approvals.
