# Supplementary material for: Validation of Functional Lumen Imaging Probe Panometry Esophageal Motility Classification Version 2.0: A Study of 805 Patients
Source: Neurogastroenterol Motil. 2025 Oct 22;37(12):e70188. doi: 10.1111/nmo.70188 (PMC12623300; doi:10.1111/nmo.70188)
Supplement: Supplementary file 1 — Appendix S1: nmo70188‐sup‐0001‐AppendixS1.docx. [file NMO-37-e70188-s001.docx]

**Supplemental Table 1 – Classification of Esophageal Motility with FLIP Panometry Version 1.0**

| **FLIP panometry CR patterns** | **Definition** | |
| --- | --- | --- |
| Normal contractile response | Repetitive antegrade contractions, defined by the rule of 6’s (Ro6s):  • ≥6 consecutive antegrade contractions of  • ≥6 cm in axial length occurring at  • 6 +/- 3 antegrade contractions per minute regular rate | |
| Borderline contractile response | • Not meeting repetitive antegrade contraction Ro6s  • Distinct antegrade contractions of at least 6-cm axial length present  • Not spastic-reactive contractile response | |
| Impaired/disordered contractile response | • No distinct antegrade contractions  • May have sporadic or chaotic contractions not meeting antegrade contractions  • Not spastic-reactive contractile response | |
| Absent contractile response | • No contractile activity in the esophageal body | |
| Spastic-reactive contractile response | Presence of any of the following features:  • Sustained occluding contractions *or*  • Sustained lower esophageal sphincter contractions *or*  • Repetitive retrograde contractions, defined by at least 6 consecutive retrograde contractions occurring at a rate of >9 contractions per minute | |
| **FLIP panometry EGJ opening classification** |  | |
| Reduced EGJ opening classification | • Esophagogastric junction distensibility index (EGJ-DI) < 2.0 mm^2^/mmHg *and*  • Maximum EGJ diameter <12 mm | |
| Borderline EGJ opening | • EGJ-DI <2.0 mm^2^/mmHg *or* maximum EGJ diameter <16 mm  • Not meeting criteria for “reduced EGJ opening” | |
| Normal EGJ opening | • EGJ-DI ≥2.0 mm^2^/mmHg *and*  • Maximum EGJ diameter ≥16 mm | |
| **FLIP panometry motility classification, v1.0** | | |
| **Motility classification** | **EGJ opening classification** | **Contractile response pattern** |
| Normal motility | • Normal | • Normal or  • Borderline |
| Weak | • Normal | • Impaired/disordered or  • Absent |
| Obstruction with normal contractile response | • Reduced | • Normal |
| Obstruction with weak contractile response | • Reduced | • Impaired/disordered or  • Absent |
| Spastic-reactive | • *Any* | • Spastic-reactive |
| Inconclusive | • Borderline | • *Any aside from spastic-reactive* |
|  | • Reduced | • Borderline |

The contractile response (CR) to distension was based on the evaluation of the functional lumen imaging probe (FLIP) study protocol including from the 50- to 70-mL fill volume. EGJ opening applied the EGJ-DI from the 60-mL FLIP fill volume and the maximum EGJ diameter from the 60- or 70-mL FLIP fill volume.

**Supplemental Table 2 – Cohort Characteristics for Site 1 (Northwestern)**

|  |  | **FLIP Panometry v2.0 Motility Classification** | | | | | | |
| --- | --- | --- | --- | --- | --- | --- | --- | --- |
|  | **Total**  n = 607 | | **Normal**  n = 124 | **Hypo-**  **contractility**  n = 65 | **Non-Spastic Obstruction**  n = 138 | **Spastic Obstruction**  n = 80 | **Possible Spasm**  n = 28 | **Possible Obstruction**  n = 172 |
| *Demographics* |  | |  |  |  |  |  |  |
| **Age, mean (SD)** | 54 (17) | | 44 (16) | 55 (15) | 51 (18) | 64 (13) | 57 (14) | 59 (15) |
| **Sex, female** | 371 (61) | | 88 (71) | 51 (78) | 58 (42) | 42 (53) | 25 (89) | 107 (63) |
| **Indication** |  | |  |  |  |  |  |  |
| Dysphagia | 485 (82) | | 89 (74) | 46 (73) | 118 (88) | 72 (90) | 18 (67) | 142 (86) |
| Reflux symptoms | 35 (5.9) | | 22 (18) | 7 (11) | 1 (0.7) | 1 (1.3) | 1 (3.7) | 3 (1.8) |
| Chest pain | 23 (3.9) | | 2 (1.7) | 3 (4.8) | 1 (0.7) | 3 (3.8) | 4 (15) | 10 (6.0) |
| Other | 48 (8.1) | | 8 (6.6) | 7 (11) | 14 (10) | 4 (5.0) | 4 (15) | 11 (6.6) |
| *Endoscopy* |  | |  |  |  |  |  |  |
| **Esophagitis** |  | |  |  |  |  |  |  |
| LA grade A | 24 (4.0) | | 9 (7.3) | 6 (9.2) | 1 (0.7) | 2 (2.5) | 0 (0) | 6 (3.5) |
| LA grade B | 13 (2.1) | | 4 (3.2) | 6 (9.2) | 0 (0) | 0 (0) | 0 (0) | 3 (1.7) |
| **Non-obstructing**  **ring** | 18 (3.0) | | 7 (5.6) | 2 (3.1) | 0 (0) | 6 (7.5) | 0 (0) | 3 (1.7) |
| **Diverticulum** | 21 (3.5) | | 0 () | 1 (1.5) | 6 (4.3) | 5 (6.3) | 4 (14) | 5 (2.9) |
| *Manometry* |  | |  |  |  |  |  |  |
| **Conclusive**  **CCv4.0 Diagnosis** |  | |  |  |  |  |  |  |
| Normal | 190 (31) | | 93 (75) | 10 (15) | 7 (5.1) | 13 (16) | 13 (46) | 54 (31) |
| IEM | 76 (13) | | 15 (12) | 20 (31) | 2 (1.4) | 4 (5.0) | 10 (36) | 25 (15) |
| Absent | 42 (6.9) | | 2 (1.6) | 32 (49) | 1 (0.7) | 2 (2.5) | 1 (3.6) | 4 (2.3) |
| DES | 6 (1.0) | | 2 (1.6) | 0 (0) | 0 (0) | 1 (1.3) | 0 (0) | 3 (1.7) |
| Hypercontractile | 22 (3.6) | | 4 (3.2) | 0 (0) | 0 (0) | 7 (8.8) | 3 (11) | 8 (4.7) |
| EGJOO | 25 (4.1) | | 2 (1.6) | 0 (0) | 9 (6.5) | 7 (8.8) | 0 (0) | 7 (4.1) |
| Type I achalasia | 67 (11) | | 0 (0) | 0 (0) | 47 (34) | 2 (2.5) | 0 (0) | 18 (10) |
| Type II achalasia | 101 (17) | | 0 (0) | 0 (0) | 56 (41) | 18 (23) | 0 (0) | 27 (16) |
| Type III achalasia | 29 (4.8) | | 0 (0) | 0 (0) | 7 (5.1) | 14 (18) | 0 (0) | 8 (4.7) |
| **Inconclusive**  **CCv4.0 Diagnosis** |  | |  |  |  |  |  |  |
| Inconclusive  EGJOO | 46 (7.6) | | 6 (4.8) | 3 (4.6) | 7 (5.1) | 12 (15) | 1 (3.6) | 17 (9.9) |
| Inconclusive | 3 (0.5) | | 0 (0) | 0 (0) | 2 (1.4) | 0 (0) | 0 (0) | 1 (0.6) |
| **HRM-EGJ**  **Morphology** |  | |  |  |  |  |  |  |
| Type I (no HH) | 381 (68) | | 68 (57) | 25 (40) | 119 (93) | 48 (72) | 14 (58) | 107 (68) |
| Type II-III (HH) | 177 (32) | | 51 (43) | 38 (60) | 9 (7.0) | 19 (28) | 10 (42) | 50 (32) |

Values represent n (%) unless otherwise specified.

CCv4.0, Chicago Classification v4.0; DES, distal esophageal spasm; EGJ, esophagogastric junction; EGJOO, esophagogastric junction outflow obstruction; HH, hiatal hernia; HRM, high-resolution esophageal manometry; IEM, ineffective esophageal motility

**Supplemental Table 3 – Cohort Characteristics for Site 2 (Baylor)**

|  |  | **FLIP Panometry v2.0 Motility Classification** | | | | | | |
| --- | --- | --- | --- | --- | --- | --- | --- | --- |
|  | **Total**  n = 198 | | **Normal**  n = 32 | **Hypo-**  **contractility**  n = 19 | **Non-Spastic Obstruction**  n = 36 | **Spastic Obstruction**  n = 17 | **Possible Spasm**  n = 34 | **Possible Obstruction**  n = 60 |
| *Demographics* |  | |  |  |  |  |  |  |
| **Age, mean (SD)** | 57 (17) | | 51 (17) | 50 (17) | 54 (18) | 64 (16) | 60 (16) | 59 (15) |
| **Sex, female** | 124 (63) | | 24 (75) | 8 (42) | 18 (50) | 9 (53) | 27 (79) | 38 (63) |
| **Indication** |  | |  |  |  |  |  |  |
| Dysphagia | 119 (60) | | 11 (34) | 11 (58) | 26 (72) | 15 (88) | 20 (59) | 36 (60) |
| Reflux symptoms | 29 (15) | | 12 (38) | 0 (0) | 3 (8.3) | 1 (5.9) | 5 (15) | 8 (13) |
| Chest pain | 12 (6.1) | | 3 (9.4) | 2 (11) | 2 (5.6) | 0 (0) | 2 (5.9) | 3 (5.0) |
| Other | 41 (21) | | 6 (19) | 6 (32) | 6 (17) | 1 (5.9) | 9 (26) | 13 (22) |
| *Endoscopy* |  | |  |  |  |  |  |  |
| **Esophagitis** |  | |  |  |  |  |  |  |
| LA grade A | 6 (3.0) | | 1 (3.1) | 1 (5.3) | 0 (0) | 0 (0) | 3 (8.8) | 1 (1.7) |
| LA grade B | 4 (2.0) | | 0 (0) | 1 (5.3) | 0 (0) | 0 (0) | 1 (2.9) | 2 (3.3) |
| **Non-obstructing**  **ring** | 3 (1.5) | | 1 (3.1) | 0 (0) | 1 (2.8) | 1 (5.9) | 0 (0) | 0 (0) |
| **Diverticulum** | 1 (0.5) | | 0 (0) | 0 (0) | 0 (0) | 0 (0) | 1 (2.9) | 0 (0) |
| *Manometry* |  | |  |  |  |  |  |  |
| **Conclusive**  **CCv4.0 Diagnosis** |  | |  |  |  |  |  |  |
| Normal | 74 (37) | | 18 (56) | 11 (58) | 3 (8.3) | 1 (5.9) | 20 (59) | 21 (35) |
| IEM | 12 (6.1) | | 1 (3.1) | 2 (11) | 0 (0) | 0 (0) | 2 (5.9) | 7 (12) |
| Absent | 3 (1.5) | | 0 (0) | 2 (11) | 0 (0) | 0 (0) | 0 (0) | 1 (1.7) |
| DES | 3 (1.5) | | 0 (0) | 0 (0) | 0 (0) | 0 (0) | 0 (0) | 3 (5.0) |
| Hypercontractile | 7 (3.5) | | 0 (0) | 0 (0) | 2 (5.6) | 1 (5.9) | 1 (2.9) | 3 (5.0) |
| EGJOO | 3 (1.5) | | 0 (0) | 0 (0) | 0 (0) | 0 (0) | 0 (0) | 3 (5.0) |
| Type I achalasia | 5 (2.5) | | 0 (0) | 0 (0) | 3 (8.3) | 0 (0) | 1 (2.9) | 1 (1.7) |
| Type II achalasia | 31 (16) | | 0 (0) | 1 (5.3) | 20 (56) | 4 (24) | 0 (0) | 6 (10) |
| Type III achalasia | 8 (4.0) | | 0 (0) | 0 (0) | 6 (17) | 2 (12) | 0 (0) | 0 (0) |
| **Inconclusive**  **CCv4.0 Diagnosis** |  | |  |  |  |  |  |  |
| Inconclusive  EGJOO | 52 (26) | | 13 (41) | 3 (16) | 2 (5.6) | 9 (53) | 10 (29) | 15 (25) |
| Inconclusive | 0 (0) | | 0 (0) | 0 (0) | 0 (0) | 0 (0) | 0 (0) | 0 (0) |
| **HRM-EGJ**  **Morphology** |  | |  |  |  |  |  |  |
| Type I (no HH) | 128 (65) | | 11 (34) | 12 (63) | 30 (83) | 11 (65) | 20 (59) | 44 (73) |
| Type II-III (HH) | 70 (35) | | 21 (66) | 7 (37) | 6 (17) | 6 (35) | 14 (41) | 16 (27) |

Values represent n (%) unless otherwise specified.

CCv4.0, Chicago Classification v4.0; DES, distal esophageal spasm; EGJ, esophagogastric junction; EGJOO, esophagogastric junction outflow obstruction; HH, hiatal hernia; HRM, high-resolution esophageal manometry; IEM, ineffective esophageal motility

**Supplemental Table 4** **– Characteristics for Inconclusive Manometry**

|  |  | **FLIP Panometry v2.0 Motility Classification** | | | | | |
| --- | --- | --- | --- | --- | --- | --- | --- |
|  | **Total**  n = 101 | **Normal**  n = 19 | **Hypo-**  **contractility**  n = 6 | **Non-Spastic Obstruction**  n = 11 | **Spastic Obstruction**  n = 21 | **Possible Spasm**  n = 11 | **Possible Obstruction**  n = 33 |
| *Demographics* |  |  |  |  |  |  |  |
| **Age, mean (SD)** | 61 (14) | 53 (15) | 55 (18) | 54 (18) | 67 (11) | 66 (13) | 64 (10) |
| **Sex, female** | 67 (66) | 14 (74) | 5 (83) | 6 (55) | 14 (67) | 7 (64) | 21 (64) |
| **Indication** |  |  |  |  |  |  |  |
| Dysphagia | 71 (70) | 10 (53) | 4 (67) | 10 (91) | 16 (76) | 6 (55) | 25 (76) |
| Reflux symptoms | 13 (13) | 7 (37) | 0 (0) | 0 (0) | 2 (9.5) | 1 (9.1) | 3 (9.1) |
| Chest pain | 7 (6.9) | 1 (5.3) | 0 (0) | 0 (0) | 1 (4.8) | 1 (9.1) | 4 (12) |
| Other | 11 (11) | 1 (5.3) | 2 (33) | 1 (9.1) | 2 (9.5) | 4 (36) | 1 (3.0) |
| *Endoscopy* |  |  |  |  |  |  |  |
| **Esophagitis** |  |  |  |  |  |  |  |
| LA grade A | 1 (1.0) | 0 (0) | 0 (0) | 0 (0) | 1 (4.8) | 0 (0) | 0 (0) |
| LA grade B | 1 (1.0) | 0 (0) | 0 (0) | 0 (0) | 0 (0) | 0 (0) | 1 (3.0) |
| **Non-obstructing**  **ring** | 2 (2.0) | 1 (5.3) | 0 (0) | 0 (0) | 1 (4.8) | 0 (0) | 0 (0) |
| **Diverticulum** | 3 (3.0) | 0 (0) | 0 (0) | 0 (0) | 1 (4.8) | 1 (9.1) | 1 (3.0) |
| *Manometry* |  |  |  |  |  |  |  |
| **CCv4.0**  **Diagnosis** |  |  |  |  |  |  |  |
| Inconclusive  EGJOO | 98 (97) | 19 (100) | 6 (100) | 9 (82) | 21 (100) | 11 (100) | 32 (97) |
| Inconclusive | 3 (3.0) | 0 (0) | 0 (0) | 2 (18) | 0 (0) | 0 (0) | 1 (3.0) |
| **HRM-EGJ**  **Morphology** |  |  |  |  |  |  |  |
| Type I (no HH) | 62 (61) | 7 (37) | 5 (83) | 9 (82) | 12 (57) | 7 (64) | 22 (67) |
| Type II-III (HH) | 32 (32) | 11 (58) | 1 (17) | 1 (9) | 7 (33) | 4 (36) | 8 (24) |

Values represent n (%) unless otherwise specified.

CCv4.0, Chicago Classification v4.0; DES, distal esophageal spasm; EGJ, esophagogastric junction; EGJOO, esophagogastric junction outflow obstruction; HH, hiatal hernia; HRM, high-resolution esophageal manometry; IEM, ineffective esophageal motility
